# Problem opioid use and HIV primary care engagement among hospitalized people who use drugs and/or alcohol

**DOI:** 10.1186/s13722-020-00192-9

**Published:** 2020-06-19

**Authors:** Lacey Critchley, Adam Carrico, Natalie Gukasyan, Petra Jacobs, Raul N. Mandler, Allan E. Rodriguez, Carlos del Rio, Lisa R. Metsch, Daniel J. Feaster

**Affiliations:** 1 Department of Public Health Sciences, Miller School of Medicine, University of Miami, 1120 NW 14th Street, Miami, FL 33136 USA; 2 Behavioral Pharmacology Research Unit, Johns Hopkins Bayview Medical Center, Baltimore, MD USA; 3 Center for the Clinical Trials Network, National Institute on Drug Abuse, National Institutes of Health, Bethesda, MD USA; 4 Division of Infectious Diseases, Department of Medicine, Miller School of Medicine, University of Miami, Miami, FL USA; 5 Department of Medicine, Emory University School of Medicine, Atlanta, GA USA; 6 Department of Sociomedical Sciences, Mailman School of Public Health, Columbia University, New York, NY USA

**Keywords:** Opioid, Drug use, HIV primary care, Treatment as prevention

## Abstract

**Background:**

There is growing public health concern around the potential impact of the opioid crisis on efforts to eradicate HIV. This secondary analysis seeks to determine if those who report opioids as their primary problem drug compared to those who report other drugs and/or alcohol differ in engagement in HIV primary care among a sample of hospitalized people with HIV (PWH) who use drugs and/or alcohol, a traditionally marginalized and difficult to engage population key to ending the HIV epidemic.

**Setting and participants:**

A total of 801 participants (67% male; 75% Black, non-Hispanic; mean age 44.2) with uncontrolled HIV and reported drug and/or alcohol use were recruited from 11 hospitals around the U.S. in cities with high HIV prevalence from 2012 to 2014 for a multisite clinical trial to improve HIV viral suppression.

**Methods:**

A generalized linear model compared those who reported opioids as their primary problem drug to those who reported other problem drugs and/or alcohol on their previous engagement in HIV primary care, controlling for age, sex, race, education, income, any previous drug and/or alcohol treatment, length of time since diagnosis, and study site.

**Results:**

A total of 95 (11.9%) participants reported opioids as their primary problem drug. In adjusted models, those who reported opioids were significantly less likely to have ever engaged in HIV primary care than those who reported no problem drug use (adjusted risk ratio, *ARR* = 0.84, 95% *Confidence Interval, CI* 0.73, 0.98), stimulants (*ARR* = 0.84, 95% *CI* 0.74, 0.95), and polydrug use but no alcohol (*ARR* = 0.79, 95% *CI* 0.68, 0.93). While not statistically significant, the trend in the estimates of the remaining drug and/or alcohol categories (alcohol, cannabis, polydrug use with alcohol, and [but excluding the estimate for] other), point to a similar phenomena—those who identify opioids as their primary problem drug are engaging in HIV primary care less.

**Conclusions:**

These findings suggest that for hospitalized PWH who use drugs and/or alcohol, tailored and expanded efforts are especially needed to link those who report problem opioid use to HIV primary care.

*Trial registration* This study was funded by National Institutes of Health (NIH) grant: U10-DA01372011 (*Project HOPE*—*Hospital Visit as Opportunity for Prevention and Engagement for HIV*-*Infected Drug Users*; Metsch); which is also a registered clinical trial under the Clinical Trials Network (CTN-0049). The content is solely the responsibility of the authors and does not necessarily represent the official views of the NIH.

## Background

Despite significant advancements in HIV treatment since the start of the HIV epidemic, marginalized groups continue to be disproportionately affected by the virus. People who use drugs remain one of the most difficult groups to engage and retain in HIV care and often have unsuppressed, poorly controlled HIV infection as a result of inadequate antiretroviral adherence [1, 2]. People who use drugs are also more likely to engage in risky behaviors including condomless sex [3], having multiple concurrent partners [4], and syringe-sharing for injection drug use [5, 6], putting them at increased risk for coinfection (including HCV coinfection), faster disease progression, and onward HIV transmission [5].

The opioid crisis has led to devastating health consequences in nearly every region and demographic in the U.S [6]. The rise in problem opioid use combined with injection drug use is fueling an increase in infectious disease incidence including HIV [7, 8], viral hepatitis [7], infective endocarditis [9], skin and soft-tissue infections [10], as well as other serious infections [11, 12]. The Centers for Disease Control and Prevention (CDC) estimates that 1 in 10 new HIV infections in the US are attributable to persons who inject drugs (PWID) [13]. Recent HIV outbreaks in Scott County, Indiana and Lawrence and Lowell, Massachusetts attribute injection opioid use to 13% of new cases [14, 15]. Recent clinical guidelines and policy recommendations from the Infectious Disease Society of America have also been updated to reflect increasing reports of HIV and other infectious diseases among people with problem opioid use [16].

Given the magnitude of the opioid crisis and the growing concern that it may fuel increased HIV incidence in the near future [17], it is imperative to better understand how those who report problem opioid use differ from those who report other drugs and/or alcohol in their engagement in HIV primary care. As approximately 92% of new HIV infections in the U.S. are transmitted by people who are either undiagnosed, or diagnosed but not engaged in care, understanding how opioids influence care engagement can help tailor intervention efforts that optimize the clinical and public health benefits of HIV treatment as prevention [18].

## Methods

### Setting

The Hospital Visit as Opportunity for Prevention and Engagement for HIV-infected Drug Users (Project HOPE) study enrolled people who use drugs and/or alcohol in a multi-site randomized controlled trial conducted in the National Institute on Drug Abuse (NIDA) Clinical Trials Network (CTN-0049). Participants of this study were recruited from July 2012 to January 2014 during medical hospitalizations from 11 hospitals in major urban areas across the U.S. with high HIV prevalence (Boston, MA; New York, NY; Philadelphia, PA: Baltimore, MD; Pittsburgh, PA; Chicago, IL; Atlanta, GA; Miami, FL; Birmingham, AL; Dallas, TX; and Los Angeles, CA). Follow-up was completed in April 2015, and data were locked in June 2015.

### Participants

A total of 2,291 patients were assessed for study eligibility. Participants were eligible if they (1)were inpatients with HIV infection, (2) were at least 18 years old, (3) signed a medical record release, (4) lived in the vicinity, (5) completed the baseline assessment, (6) could communicate in English, (7) provided information on where and how to locate them, (8) had functional status of 60 or higher on the Karnofsky performance scale, (9) reported or had medical records documenting any opioid (other than as directed by a physician prescription), stimulant (cocaine, ecstasy, or amphetamines), or heavy alcohol use as determined by the Alcohol Use Disorders Identification Test for Consumption (AUDIT)-C [19] within the past 12 months, and (10) met one of the following requirements: had an AIDS-defining illness; had a CD4 cell count less than 350 cells/μL at their most recent screening and a viral load of more than 200 copies/mL within 6 months; or had a CD4 cell count within 12 months that was 500 cells/μL or less and their viral load was more than 200 copies/mL (or their viral load was unknown with clinical indicators that the patient was likely to have a detectable viral load).

A total of 801 eligible patients completed the baseline assessment and were then assigned to one of three conditions (Patient Navigation, Patient Navigation with financial incentives, Treatment as Usual). Interventions were designed to help link and retain patients in HIV care as well as drug or alcohol treatment, and to help them initiate and/or maintain HIV antiretroviral therapy (ART) medication, with the ultimate goal of attaining virologic suppression. Those randomized to one of the Patient Navigation arms were offered up to 11 sessions with a patient navigator during the 6-month intervention period. Repeat assessments were conducted at six-month (n = 761) and twelve-month follow-ups (n = 752). Detailed eligibility requirements and trial results have been published previously [20].

The study sample of Project HOPE was predominantly male (67.5%), and minority (87.9%), which is reflective of the population of PWH in the U.S. Most participants reported a high school education or greater (60.6%). The average age of participants was 44.2 years (SD = 10.0). Most participants had engaged in HIV care at some point in the past (82.9%), with 50.1% on HIV medication at baseline. By the end of the study (12 months post-baseline), 90 participants had died, 26 were loss to follow up [18].

The secondary analysis presented in this paper utilizes baseline data from Project HOPE’s 801 participants. Self-reported history of HIV primary care engagement as well as reported problem drug and/or alcohol use were collected before clinical trial condition assignment.

### Measures

#### Primary problem drug

After answering detailed questions on polydrug use using the validated Addiction Severity Index (ASI)—Lite [21], participants answered the question, “Currently, which substance is the major problem?*”* Choices included: no problem drug use; alcohol; heroin; methadone (prescribed or illicit); other opiates/analgesics; barbiturates/sedatives/hypnotics/tranquilizers; cocaine; amphetamines; cannabis; hallucinogens; inhalants; and polydrug with or without alcohol (a total of 13 options). For the purposes of this secondary analysis, those who reported heroin, methadone (prescribed or illicit), or other opiates/analgesics were combined to form an “opioids” category due to the small number of study participants in each nuanced category; cocaine and amphetamines were combined into a “stimulants” category due to the small number of people who reported amphetamines; and an “other” category was created for minimally-reported groups (barbiturates/sedatives/hypnotics/tranquilizers, hallucinogens, inhalants). Thus, a total of 8 problem drug and/or alcohol categories were used for comparison in this analysis—no problem drug use, opioids, alcohol, stimulants, cannabis, polydrug use with alcohol, polydrug use but no alcohol, and other.

The “primary problem drug” measure was chosen from the study’s rich data on drug and alcohol use because it is thought that this question reflects which drug participants assess themselves to be struggling with the most and/or which drug likely has the biggest impact on their life. If participants chose a drug in the collapsed “opioids” category, it is then assumed that, even if prescribed opioids, they were not using as directed as per physician-administered prescriptions.

#### Engagement in HIV primary care

Participants answered the question, “Have you ever had HIV primary care? By HIV primary care, we mean a clinician or team of clinicians who you see in a clinic or office on a regular basis and who works with you to manage your HIV/AIDS medications, blood test results, T cell count and viral load.” This variable was dichotomized as no/yes (0/1) for those who provided an answer.

#### Covariates

Models controlled for age (measured as an integer rounded to the nearest tenth); sex (male/female); race (non Hispanic black, non Hispanic white, Hispanic, or other); education (less than high school, high school, greater than high school); health insurance (no/yes), annual income (integer), any previous drug and/or alcohol treatment (no/yes), length of time since HIV diagnosis (as an integer in months); and study site (a categorical variable of 11 study sites).

### Data analysis

Demographic variables were compared between primary problem drug and/or alcohol categories using Chi square analyses. A multivariate, generalized linear model compared those who reported opioids as their primary problem drug to those who reported other primary problem drugs and/or alcohol categories on their previous engagement in HIV primary care. The generalized linear model (with a poisson distribution for the dichotomous outcome variable and a log link function) was used to provide adjusted risk ratios, which were deemed to be more useful than a logistic regression’s adjusted odds ratios, as the outcome of interest was not a rare event. Relative risk estimates are preferred (when justified), as odds ratios are used to provide (biased) estimates of the risk ratio. All analyses were done using SAS version 9.4 [22].

## Results

Of the 801 study participants, 95 (11.9%) reported opioids (heroin n = 89, prescribed or illicit methadone n = 4, or other opiates n = 2) as their primary problem drug. Those who reported opioids as their primary problem drug (vs. those who reported any other primary problem drug and/or alcohol for simplicity) were found to be significantly: older, with a mean age of 48 (SD 8.6) vs. 44 (SD 9.9); Hispanic (21.3% vs. 9.4%) and foreign-born (15.8% vs. 6.1%); were more likely to live in what were considered the “northern” sites (New York, Boston, Philadelphia, Chicago, Pittsburgh, Los Angeles; 85.3% vs. 42.2%); to have health insurance (75.8% vs. 65.1%); to have previously engaged in drug and/or alcohol treatment of some nature (71.6% vs. 54.8%); were more likely to be unemployed (37.9% vs. 34.8%) and had less annual income, with an average of $7,286 (SD $7760) vs. $10,290 (SD $10,430); and finally, those who reported opioids as their primary problem drug had diagnosed HIV infection longer, with an average length of time since HIV diagnosis of 172.1 months (SD 100.9) vs. 142.68 months (SD 103.25). Full demographic details per each of the eight primary problem drug and/or alcohol categories are presented in Table 1.

**Table 1:** Demographic differences of participants according to reported primary problem drug

	Opioids (N = 95)		No Problem Drug Use (N = 83)		Alcohol (N = 182)		Stimulants (N = 287)		Cannabis (N = 46)		Polydrug with Alcohol (N = 62)		Polydrug no Alcohol (N = 40)		Other (N = 5)		p-value
	n	%	n	%	n	%	n	%	n	%	n	%	n	%	n	%	
Sex																	0.02^b^
Male	58	61.1	51	61.5	141	77.5	179	62.4	35	76.1	44	70.8	28	70.0	4	80.0	
Female	37	39.0	32	38.6	41	22.5	108	37.6	11	23.9	18	29.0	12	30.0	1	20.0	
Age^a^	48	8.6	43	10.8	44	10.3	45	9.0	34	9.5	43	9.6	43	9.8	39	13.3	< 0.001^b^
Race																	< 0.001^b^
NH Black	63	67.0	61	73.5	139	76.8	231	80.8	39	86.7	41	66.1	21	52.5	3	60.0	
NH white	11	11.7	12	14.5	25	13.8	25	8.7	3	6.7	11	17.7	13	32.5	0	0.0	
Hispanic	20	21.3	10	12.1	14	7.7	25	8.7	2	4.4	9	14.5	5	12.5	2	40.0	
NH other	0	0.0	0	0.00	3	1.7	5	1.8	1	2.2	1	1.6	1	2.5	0	0.0	
Education																	0.44
< High School	42	44.2	29	34.9	62	34.1	126	43.9	14	30.4	26	41.9	17	42.5	2	40.0	
High School/GED	37	39.0	29	34.9	67	36.8	88	30.7	18	39.1	21	33.9	10	25.0	1	20.0	
> High School	16	16.8	25	30.1	53	29.1	73	25.4	14	30.4	15	24.2	13	32.5	2	40.0	
Employment status																	< 0.001^b^
Working	4	4.21	15	18.1	32	17.6	23	8.0	8	17.4	8	12.9	3	7.5	0	0.0	
Unemployed	36	37.9	28	33.7	64	35.2	85	29.6	22	47.8	27	43.6	16	40.0	2	40.0	
Disabled	46	48.4	38	45.8	79	43.4	172	59.9	14	30.4	27	43.6	20	50.0	3	60.0	
Other	9	9.5	2	2.4	7	3.9	7	2.4	2	4.4	0	0.0	1	2.5	0	0.0	
Annual income, $1000s^a^	7.3	7.8	16.7	40.7	12.7	12.5	9.9	9.6	10.4	10.4	75.3	8.8	7.4	7.3	9.8	3.6	0.02^b^
Marital Status																	0.32
Married/cohabitating	13	13.7	11	13.3	25	13.7	32	11.2	3	6.5	4	6.5	2	5.0	0	0.0	
Widowed/divorced/separated	25	26.3	20	24.1	34	18.7	75	26.1	6	13.0	13	21.0	7	17.5	1	20.0	
Never married	57	60.0	52	62.7	123	67.6	180	62.7	37	80.4	45	72.6	31	77.5	4	80.0	
Health insurance	72	75.8	60	73.2	119	65.4	204	72.1	23	50.0	30	48.4	22	55.0	3	75.0	< 0.001^b^
Unstably housed																	
Most of last 6 months	24	25.5	17	21.8	37	20.4	81	28.4	10	21.7	18	29.5	7	17.5	1	20.0	0.49
Any of last 6 months	42	44.2	18	22.5	58	32.4	119	42.2	17	37.8	28	45.9	12	30.8	4	80.0	0.005^b^
Incarcerated																	
Last 6 months	16	17.0	10	12.1	21	11.5	50	17.4	7	15.2	8	13.1	9	22.5	0	0.0	0.47
Ever	81	86.2	54	65.1	130	71.4	236	82.2	29	63.0	52	85.3	35	87.5	3	60.0	< 0.001^b^
US born	80	84.2	79	95.2	166	91.2	274	95.5	43	93.5	58	93.6	38	95.0	5	100.0	0.027^b^
US geography																	<.001^b^
North	81	85.3	58	69.9	90	49.5	117	40.8	18	39.1	15	24.2	17	42.5	5	100.0	
South	14	14.7	25	30.1	92	50.6	170	59.2	28	60.9	47	75.8	23	57.5	0	0.0	
Food insecure	56	59.0	69	83.1	127	69.8	177	61.7	27	58.7	31	50.0	25	62.5	4	80.0	0.001^b^
Previous drug/alcohol treatment	68	71.6	34	41.0	83	45.9	187	65.2	7	15.2	37	59.7	24	60.0	2	40.0	< 0.001^b^
Length of time since HIV diagnosis in months^a^	172.1	100.9	158.3	100.4	123.0	103.0	162.2	102.3	97.6	83.2	136.2	98.9	153.0	109.0	160.6	126.3	< 0.001^b^

Denominators vary due to missing data

^a^ Mean and standard deviation presented; ^b^ Denotes statistical significance at α= 0.05

### Main findings

Results for the generalized linear model are presented in Table 2. In models adjusted for age, sex, race, education, health insurance, income, any previous drug and/or alcohol treatment, length of time since HIV diagnosis, and study site, those who reported opioids as their primary problem drug were significantly less likely to have ever engaged in HIV primary care than those who reported no problem drug use (*ARR* = 0.84, 95% *CI* 0.73, 0.98), stimulants (*ARR* = 0.84, 95% *CI* 0.74, 0.95), or polydrug use but no alcohol (*ARR* = 0.79, 95% *CI* 0.68, 0.93). These results suggest that those reporting opioids as their primary problem drug are 16- to 21- percent less likely to have ever engaged in HIV primary care. While not statistically significant, the trend in the estimates of the remaining drug and/or alcohol categories (excluding the “other” drug category) point to a similar phenomena—those who identify opioids as their primary problem drug are engaging in HIV primary care less.

**Table 2:** Differences in ever HIV primary care engagement among those who reported opioids as their primary problem drug vs. those who reported other primary problem drug and/or alcohol categories

	Opioids vs. no problem drug	Opioids vs. alcohol	Opioids vs. stimulants	Opioids vs. cannabis	Opioids vs. polysubstance with alcohol	Opioids vs. polysubstance no alcohol	Opioids vs. other
Engagement in HIV primary care	ARR = 0.84 (95% CI 0.73, 0.98)^a^	ARR = 0.96 (95% CI 0.84, 1.09)	ARR = 0.84 (95% CI 0.74, 0.95)^a^	ARR = 0.90 (95% CI 0.70, 1.15)	ARR = 0.86 (95% CI 0.72, 1.03)	ARR = 0.79 (95% CI 0.68, 0.93)^a^	ARR = 1.24 (95% CI 0.64, 2.40)

ARR, Adjusted Risk Ratio (controlled for age, sex, race, education, health insurance, income, any previous substance use treatment, length of time since HIV diagnosis, and study site); 95% CI 95% confidence interval; ^a^ Denotes statistical significance at α= 0.05

## Discussion

The key finding of this secondary analysis is that hospitalized patients with uncontrolled HIV infection who report opioids as their primary problem drug were significantly less likely to have ever been engaged in HIV primary care compared to those who report other primary problem drugs and/or alcohol. Notably, and perhaps most importantly, this difference exists despite the fact that this group reported having known of their HIV diagnoses for significantly longer periods of time (an average of over 14 years compared to an average of just under 12 years in other categories) and having health insurance, suggesting many missed opportunities for linkage to care.

These findings suggest that while engaging people in care after HIV diagnosis is critical for everyone, there may be additional barriers to care to consider when it comes to those who identify with having problem opioid use. These additional barriers to care can vary according to type of opioid usage, route of administration, as well as specific systemic and structural barriers related to problem opioid use. Much of the published literature related to PWH with problem opioid use has focused on PWID in particular, who face increased systemic and structural barriers to care stemming from ongoing criminalization of injection drug use [23, 24]. PWID are often subject to harmful policies including detention in centers, jails, and prisons that do not provide evidence-based treatment and the police use of registries for people who use drugs [23]. PWID also have higher rates of unstable housing and social syndemics leading to more chaotic, stressful lives for which drug use may be a coping mechanism [25]. Likewise, stigmatization, both for illicit drug use and HIV status, is another well-known barrier to HIV care that perpetuates the cycle of drug use and HIV transmission [26].

Lack of integrated care, however, is the most significant, tangible and well-documented structural barrier to both HIV care and drug and alcohol treatment, as many addiction professionals currently do not link patients to HIV care, and vice versa [27]. Given the multitude of needs PWH who have problem drug use of any kind face, if only part of the problem is addressed, success in any major health outcome is unlikely. Multidimensional, integrated interventions are needed for these traditionally marginalized and difficult to engage populations. As supported by recent literature [8, 28–31], prevention and intervention efforts should expand to places outside traditional HIV primary care settings—drug treatment centers, and in particular those that provide medications for opioid use disorder (MOUD); syringe service programs (SSPs), pain management clinics, and other places where opioids are prescribed may be key locations for linkage to HIV care. Likewise, efforts to begin MOUD (and other drug and/or alcohol treatment) while in hospital or in HIV primary care are also critical and would have been particularly useful for this study’s population. Unless PWH with problem opioid use are on MOUD, they are unlikely to get well-connected to care and adherent with antiretroviral therapy (ART) or curtail high-risk behaviors. National efforts to eradicate HIV will depend on these integrated opportunities to link high-risk, traditionally difficult to engage populations to quality HIV care as well as drug treatment.

## Limitations

There are limitations of this secondary analysis. First, the generalizability is limited to hospitalized people with self-reported problem drug and/or alcohol use who also have uncontrolled HIV infection, the selected demographic for the parent study. While generalization is limited, this is an important, traditionally marginalized population that is difficult to engage in HIV care. Second, this secondary analysis is limited to self-report data. To this regard, the “primary problem drug” categorization excluded some participants who used opioids but did not identify opioids as their primary problem drug. Also, categories had to be collapsed for effective statistical analysis, including the “opioids” category, which, if able to be separated with sufficient participants in each group (for statistical modeling purposes) could provide valuable nuanced information. The “stimulants” category was also collapsed due to the small number of participants in the amphetamines subcategory. It is suspected that stimulant type and use may differ by region, with cocaine being a predominate problem in the Eastern U.S. sites and methamphetamine use in the Western U.S. sites; HIV care engagement associations may be affected as a result. Despite these statistical limitations, we have justified using the “primary problem drug” measure with some categories collapsed, as the model is able to clearly categorize study participants (with no category overlap) on the drug they see as most impacting their lives, which is incredibly meaningful from an intervention perspective. Likewise, it is important to note that route of administration for each self-reported problem drug category was not collected relative to each primary problem drug and/or alcohol category (route and usage data was collected for all the drug categories, but these findings showed a lot of overlapping polydrug and alcohol use but did not resemble the breakdown of self-reported “primary problem drug” categories). Route and use may affect the findings and if more nuanced data were available, would be useful in interpretation. Another important point for consideration is that the model used for analysis adjusted for “any previous drug and/or alcohol treatment”; it is understood that those who had ever received treatment for their problem opioid use (including methadone treatment, which was also a response option for problem opioid use) are very different than participants with active heroin/illicit fentanyl use. Again, the distributions in the data subcategories limited the ability to further analyze and explain this important nuance. Finally, as with all cross-sectional research, a temporal effect between predictor, primary problem drug and/or alcohol category, and outcome, ever engagement in HIV primary care, cannot be attained.

## Conclusions

With increasing concern regarding the potential impact of the opioid crisis on the HIV epidemic, this secondary analysis helps identify a need for interventions to target HIV primary care engagement among hospitalized people who report problem opioid use. Findings support the need for a coordinated public health response by HIV professionals in primary care settings as well as those in hospitals, pain management specialists, and drug and alcohol treatment providers to optimize linkage to and retention of people with problem opioid use in HIV care.

## Data Availability

The datasets during and/or analyzed during the current study available from the corresponding author on reasonable request.

## Abbreviations

ARR: Adjusted risk ratio
AIDS: Acquired Immune Deficiency Syndrome
ART: Antiretroviral therapy
ASI: Addiction Severity Index
AUDIT-C: Alcohol Use Disorders Identification Test
CDC: Centers for Disease Control and Prevention
CI: Confidence Interval
CTN: Clinical Trials Network
HCV: Hepatitis C Virus
HIV: Human Immunodeficiency Virus
HOPE: Hospital Visit as Opportunity for Prevention and Engagement for HIV-Infected Drug Users
MOUD: Medications for opioid use disorder
NIDA: National Institute on Drug Abuse
NIH: National Institutes of Health
PWH: People with HIV
PWID: Persons who inject drugs
SD: Standard deviation
SSPs: Syringe Service Programs
